# Lymph Node Metastasis and Extrathyroidal Extension in Papillary Thyroid Microcarcinoma in Cyprus: Suspicious Subcentimeter Nodules Should Undergo FNA When Multifocality is Suspected

**DOI:** 10.1155/2020/3567658

**Published:** 2020-03-24

**Authors:** Christos Papaioannou, Demetris Lamnisos, Katerina Kyriacou, Theodoros Lyssiotis, Vasilis Constantinides, Savvas Frangos, Aliki Economides, Panayiotis A. Economides

**Affiliations:** ^1^Barts and the London School of Medicine and Dentistry, Queen Mary University of London, London, UK; ^2^European University Cyprus, Engomi, Nicosia, Cyprus; ^3^Histopathology and Cytology Medical Center, Nicosia, Cyprus; ^4^Department of Endocrine Surgery, Evangelistria Medical Center, Engomi, Nicosia, Cyprus; ^5^Bank of Cyprus Oncology Center, Strovolos, Nicosia, Cyprus; ^6^Thyroid & Endocrinology Center, Engomi, Nicosia, Cyprus

## Abstract

**Objective:**

To determine the prevalence of lymph node (LN) metastasis and extrathyroidal extension (ETE) in patients with papillary thyroid microcarcinoma (PTMC) in Cyprus and to evaluate the role of preoperative ultrasound (U/S) examination.

**Methods:**

A retrospective study of 102 patients who underwent thyroidectomy for PTMC in a 2-year period. Preoperatively, all patients had a thyroid and neck U/S examination with LN mapping. Tumor size according to the largest diameter, number of foci, LN metastasis, and ETE data was collected from the histopathological report and was compared to the preoperative U/S reports.

**Results:**

LN metastasis was present in 23.5% of patients. 15.7% had central, 3.9% had lateral, and 3.9% had both central and lateral LN metastasis. ETE was present in 27.5% of patients. 21.6% had multifocal disease, and in this group, 40.9% had LN metastasis and 36.4% had ETE. Multifocality (*p* = 0.03), size of tumor (*p* = 0.05), and ETE (*p* ≤ 0.001) were significantly associated with LN metastasis. The prevalence of LN metastasis in multifocal PTMC ≤5 mm was the same with multifocal PTMC >5 mm. The preoperative U/S sensitivity for the suspicious lateral neck and central LN was 100%, and the specificity was 100%. The preoperative U/S sensitivity for nodules suspicious for ETE was 53.6%, and the specificity was 100%.

**Conclusion:**

The presence of LN metastasis and ETE in our PTMC patients in Cyprus is frequent. Neck U/S mapping is a highly reliable and accurate tool in identifying metastatic nodes. LN metastasis is associated with ETE and multifocality. Suspicious subcentimeter nodules should undergo FNA irrespective of size when multifocality is suspected.

## 1. Introduction

There is a high prevalence of papillary thyroid carcinoma worldwide with an increasing incidence attributed to the increased diagnosis of papillary thyroid microcarcinoma (PTMC) [1, 2]. PTMC are tumors less than or equal to 10 mm along the greatest diameter [3] and are most often incidentally identified during routine thyroid ultrasonography [4, 5] with the major risk factors being a positive family history and exposure to ionising radiation [6]. Their mortality rate is less than 1%, and they have an excellent prognosis [7]. In a 2015 study analysing thyroid nodules of patients in the island of Cyprus, 14.3% of those were found to be malignant [8] with thyroid cancer being the second most common type of cancer in Cypriot female patients [9]. In a cohort of low-intermediate risk papillary thyroid carcinoma patients undergoing radioiodine ablation in a referral oncology center in Cyprus, almost one quarter had cervical LN metastasis [10].

The management of PTMC is controversial and debatable. The American Thyroid Association (ATA), the American Association of Clinical Endocrinologists (AACE), the European Thyroid Association (ETA), and British Thyroid Association (BTA) guidelines advice against ultrasound-guided fine needle aspiration biopsy (USgFNA) in thyroid nodules less or equal to 10 mm provided that there are no metastatic LN or suspicion of ETE [11–14]. AACE recommends against FNA in incidental thyroid lesions with a diameter <5 mm as they are considered to have a lower risk of aggressive features, and there is an increased risk of inadequate sampling [13]. In cytologically proven thyroid microcarcinomas, presumed to be low-risk PTMC, active surveillance is currently considered as an alternative to surgery, and close observation may be advised [11, 15].

Total thyroidectomy or hemithyroidectomy is the current standard of practice with the latest ATA guidelines favouring towards hemithyroidectomy for PTMC without aggressive features [11, 16]. However, many surgeons suggest total thyroidectomy as the first option as more metastatic LN can be identified during total thyroidectomy with central LN dissection [16–18]. In addition, total thyroidectomy improves postoperative follow-up surveillance through the use of serum thyroglobulin and decreases the risk of reoccurrence [16]. Furthermore, when indicated, it facilitates the use of postoperative radioactive iodine ablation [19].

Central LN metastasis can occur in up to 29.3% and lateral lymph node metastasis in 3.7–5.6% of PTMC patients [20–22], and this is associated with locoregional re-occurrence and adverse outcomes [23, 24]. ETE is another risk factor that can affect the decision for total vs. hemithyroidectomy [16, 25]. ETE can occur in 28.0–40.3% of PTMC patients [26, 27] and is associated with increased likelihood of central, lateral LN metastasis, and increased tumor size and should be treated more aggressively [28, 29].

Ultrasonography has a major role in preoperatively evaluating the thyroid gland and the central and lateral neck LN [30–34]. Ultrasonography assesses tumor extent, the probability of ETE [35], and the location of metastatic nodes. The specificity of US to determine metastatic nodes in PTMC patients ranges from 80 to 95% in both central and lateral compartments of the neck [36]. Ultrasound-guided fine needle aspiration biopsy of the suspicious lymph nodes with thyroglobulin level determination from the aspirate is used to confirm malignancy [11, 12].

Our study is aimed to analyse the prevalence of central, lateral metastatic LN, and ETE in patients with PTMC in Cyprus and to examine the role of thyroid and neck U/S in the preoperative setting.

## 2. Methods

The records of 102 patients diagnosed with papillary thyroid microcarcinoma between a two-year period (January 2016 to December 2017) at the Thyroid & Endocrinology Center in Nicosia, Cyprus, were retrospectively reviewed. Preoperatively, all patients underwent a clinical evaluation, a thyroid ultrasound examination, U/S-guided FNA, and an U/S of the neck/LN mapping with a detailed diagram [37]. U/S was performed by an endocrinologist experienced in thyroid and neck U/S by a GE Logiq E9 system. LN was defined suspicious based on a rounded shape (ratio of short axis to long axis >0.5), peripheral hypervascularity by color Doppler examination, the presence of calcifications, cystic change, and heterogeneous texture [36]. When the nodule was peripherally located and focally abutting or contacting the thyroid capsule, the suspicion of ETE was noted in the U/S report/diagram [38, 39].

All data including age, gender and family history were recorded. The presence or absence of autoimmune thyroid disease (Hashimoto's thyroiditis and Graves' disease) was also recorded and was based on a combination of detailed history taking, clinical and ultrasonographic examination, and the presence of positive thyroid autoantibodies. Tumor size according to the largest diameter, number of foci, ETE, and LN metastasis data was collected from the histopathological report and was compared to the preoperative U/S reports. Minimal ETE was defined as microscopic tumor extension to the extrathyroidal fat, whereas gross ETE was defined as macroscopic extension to the strap muscles. In cases of unifocality, tumor size is the size in mm of the maximum diameter of the tumor. In cases of multifocality (≥2 tumor foci in the same or different lobes including the isthmus), total tumor size was defined as the sum of sizes in mm of the maximum diameter of all tumors.

All 102 patients in this cohort underwent total thyroidectomy by experienced surgeons. Ninety-eight patients had central lymph node dissection (CLND). Eight patients underwent CLND for suspicious central LN on U/S and the other 90 underwent prophylactic CLND. Four patients did not undergo CLND; however, in none of these patients suspicious central LN were seen in the preoperative U/S. Ten patients underwent lateral LN dissection; eight were based on suspicious U/S findings and/or malignant cytology/Tg washout from the LN.

The study protocol was formally submitted for approval to the Cyprus National Bioethics Committee, which advised us that as this was a retrospective record analysis, ethical clearance was not warranted. Patient identities and personal data were not revealed and were kept fully confidential throughout the study analysis.

## 3. Statistical Analysis

The following factors were examined as possible factors associated with LN metastasis and ETE: gender, age (<55 vs ≥55 years old), family history, multifocality, Hashimoto's thyroiditis, Grave's disease, tumor size, and total tumor size. The tumor size was treated as a continuous measurement (in mm) and as binary categorical variable with two categories (≤5 mm vs >5 mm). These factors were analyzed by a series of univariable logistic regression models that consider each factor separately. The association of LN metastasis and ETE with each factor was expressed in OR (odds ratios) and 95% CI (confidence intervals). Statistical analyses were performed using the statistical package SPSS 20, and statistical significance was set at *p* < 0.05.

## 4. Results

A total of 102 patients with PTMC were enrolled. Patient and disease characteristics are shown in Table 1. There were 81 females (80.4%) and 21 males (20.6%). 24/102 patients (23.5%) had lateral and/or central LN metastasis; 16/102 (15.7%) had central (level VI) LN metastasis; and 4/102 (3.9%) had lateral LN metastasis (levels II, III and IV). 4/102 (3.9%) patients had both. 9/20 (45.0%) patients with central LN metastasis and 3/8 (37.5%) patients with lateral LN metastasis had micrometastatic disease (metastatic focus <2 mm). No data regarding the size of the metastatic focus were available in 2/8 (25.0%) patients with metastatic lateral LN. The level of LN metastasis is shown in Table 2. Twenty-eight patients (27.5%) had ETE. 19/28 (67.9%) patients with ETE had minimal ETE, whereas 9/28 (32.1%) patients had gross ETE.

47/102 (46.1%) patients had tumor size less than or equal to 5 mm; in this group, 4/47 (8.5%) had central lymph node metastasis; 1/47 (2.1%) had lateral lymph node metastasis, and 3/47 6.4%) had both central and lateral lymph node metastasis. 9/47 (19.1%) of these patients had ETE. 55/102 (53.9%) patients had tumor size more than 5 mm; in this group, 12/55 (21.8%) had central lymph node metastasis; 3/55 (5.5%) had lateral lymph node metastasis; and 1/55 (1.8%) had both central and lateral lymph node metastasis. 19/55 (34.5%) of these patients had ETE.

There were 22 patients (21.6%) with multifocal disease, and Table 3 describes the number and size of the foci and the presence or absence of LN metastasis and ETE. Multifocality was suspected on the preoperative U/S in 18 patients (81.8%). 5/22 (22.7%) had central LN metastasis; 1/22 (4.5%) had lateral LN metastasis; and 3/22 (13.6%) had central and lateral LN metastasis. Micrometastatic disease was present in 2/8 (25.0%) patients with central LN metastasis and in 1/4 (25.0%) patient with lateral LN metastasis. No data were available regarding the size of the metastatic foci in 2/4 (50.0%) patients with lateral LN metastasis. 8/22 (36.4%) patients with multifocal disease had ETE. 5/8 (62.5%) had minimal ETE, whereas 3/8 (37.5%) had gross ETE.

80/102 (21.6%) had unifocal disease; in this group, 11/80 (13.8%) had central LN metastasis; 3/80 (3.8%) had lateral LN metastasis; and 1/80 (1.3%) had central and lateral LN metastasis. 20/80 (25.0%) patients had ETE.

Tables 4 and 5 show the results of the analysis of the association between different characteristics of the patients and the LN and ETE. Multifocality (*p* = 0.03), size of tumor (*p* = 0.05), total tumor size (*p* = 0.03), and ETE (*p* ≤ 0.001) were significantly associated with LN metastasis. Tumor size (*p* = 0.01), total tumor size (*p* = 0.04), and LN metastasis (*p* ≤ 0.001) were significantly associated with ETE. No association was seen between LN metastasis or ETE and family history, Hashimoto's thyroiditis, or Graves' disease.

Stratifying the sample by multifocality (Table 6) indicated that the association of tumor size with LN metastasis was different in unifocal and multifocal patients (*p* value for interaction of multifocality and tumor size was equal to 0.03). In the multifocal group, 44.4% of patients with a maximum tumor diameter ≤5 mm had LN metastasis, whereas 38.5% of the patients with maximum tumor diameter >5 mm had LN metastasis. In the unifocal group, only 10.5% patients with maximum diameter ≤5 mm had LN metastasis, whereas 38.5% of the patients with maximum tumor diameter >5 mm had LN metastasis. The association of tumor size with ETE was not statistically different in the unifocal and multifocal group (*p* value for interaction of multifocality, and tumor size was equal to 0.10), although the prevalence of ETE in the multifocal group was 33.3% in a tumor size ≤5 mm and 38.5% in a tumor size >5 mm. In the unifocal group, the prevalence of ETE was 15.8% in patients with maximum diameter ≤5 mm, whereas it was 33.3% in patients with maximum tumor diameter >5 mm.

Preoperative U/S identified all 8 patients with histologically confirmed lateral LN metastasis (sensitivity 100% and specificity 100%). All 8 patients with ultrasonographically suspicious central LN were also histologically confirmed to have central LN metastasis (sensitivity 100% and specificity 100%). The other 12 patients with central LN metastasis did not have ultrasonographically suspicious or enlarged LN, and in these patients, the size of the metastatic foci ranged between 1 and 3 mm in the histopathological examination. In retrospect, these involved small nodes, although not preoperatively characterized as suspicious, were noted and drawn in the U/S diagram. Preoperative U/S was suspicious for ETE in 15 patients with histologically confirmed ETE (sensitivity 53.6% and specificity 100%).

## 5. Discussion

The goal of this study was to determine the prevalence of LN metastasis and ETE in a cohort of patients with PTMC in Cyprus and to evaluate the role of the preoperative thyroid and neck U/S with LN mapping in assessing these patients.

The prevalence of central LN metastasis in our study is 19.6%. This rate is higher than that of the study by Bradley et al. [40] who showed a 7% prevalence and lower than that of the study by Li et al. who showed a 29.3% prevalence in PTMC patients who underwent prophylactic CLND [20]. CLND alone, without preoperatively examining the lateral neck, may miss “skip metastasis” where metastasis occurs first in the lateral compartment [40, 41]. Skip metastasis was shown in 3.9% of our patients, and this rate is higher compared to Zheng et al. [42] where skip metastasis was observed only in 1.2% of PTMC patients. Our results are similar to a study by Kwak et al. who showed a rate of lateral metastasis of 3.7% in their cohort [21]. However, Luo et al. showed a rate of 5.6% of lateral LN metastasis, and this was associated with multifocality and ETE [22]. Our results confirmed that U/S mapping is a highly reliable and accurate tool in identifying metastatic LN and guiding the surgeon for precise neck dissection. In our study, all lateral metastatic neck LN were identified preoperatively, and our high sensitivity and specificity are in agreement with other published studies [43].

Our ETE prevalence was 27.5%, and this is similar to that of a study by Lee et al. who showed a prevalence of 28% in a series of PTMC patients [26]. Kwak et al., however, showed an ETE prevalence of 40.3% in their PTMC cohort [27], and Zheng et al. showed an even higher prevalence of 65.5% [42]. Our findings showed that the presence of ETE is associated with bigger tumor size and LN metastasis and this was also illustrated by Youngwirth et al. who showed that ETE is strongly associated with metastatic LN [28]. Preoperative neck U/S is also a useful diagnostic tool in evaluating ETE preoperatively. In this regard, our study showed a moderate sensitivity but a high specificity, and this is in agreement with findings from other published studies [27, 44].

Multifocal disease was present in 21.6% of our patients, and this group had a high prevalence of aggressive features with 36.4% of those having ETE and 40.9% of those having lymph node metastasis in either central and/or lateral neck compartments. Our findings agree with those given by Zheng et al. who showed that multifocality is significantly associated with central LN metastasis in PTMC and that this may indicate higher tumor aggressiveness [45]. In our study, the rate of LN metastasis in multifocal tumors with a maximum diameter ≤5 mm was 44.4%, and this was higher to the rate in multifocal patients with a maximum diameter >5 mm. In addition, the high prevalence of ETE even in multifocal PTMC <5 mm suggest that patients with multifocal disease should be managed more aggressively. In the current study, neck ultrasound was highly accurate in the evaluation of multifocality in the preoperative setting.

Our study has some limitations. First, this is a retrospective study of a single institution, which limits the number of cases of patients with PTMC and as our study group is specific our results may not be representative of the entire population of the island. Second, the lateral compartment of the neck was dissected only when there were suspicious or malignant findings. Thus, micrometastatic lateral LN may have been missed. Nonetheless, to the best of our knowledge, this is the first study in Cyprus examining the role of the preoperative U/S examination in patients with PTMC.

In conclusion, the presence of LN metastasis and ETE in our cohort of PTMC patients in Cyprus is frequent. LN metastasis is associated with ETE and multifocality, and this is consistent with previous studies. An unexpectedly high prevalence of aggressive features was observed in multifocal “small” PTMCs less than 5 mm. We propose that multifocality should be considered as a significant risk factor, similar to ETE and LN metastasis, when deciding whether to proceed to USgFNA in subcentimeter nodules and even in <5 mm lesions. As the practice of active surveillance of small “low-risk” lesions gains more acceptance, the careful and detailed U/S examination is becoming even more critical.

## Figures and Tables

**Table 1:** Patient and disease characteristics.

Characteristics	Categories	Frequency (%)
Gender	Male	21 (20.6)
	Female	81 (80.4)
Age	<55	79 (77.5)
	≥55	23 (22.5)
Family history	Yes	15 (14.7)
	No	87 (85.3)
Multifocality	Yes	22 (21.6)
	No	80 (79.4)
Hashimoto's thyroiditis	Yes	32 (31.4)
	No	70 (69.6)
Grave's disease	Yes	3 (2.9)
	No	99 (97.1)
Tumor size (mm)		Mean (sd)
		5.94 (0.22)
Tumor size	≤5 mm	47 (46.1)
	>5 mm	55 (53.9)
ETE	Yes	28 (27.5)
	No	74 (72.5)
LN metastasis	Yes	24 (23.5)
	No	78 (77.5)
Level LN metastasis	Central	16 (15.7)
	Lateral	4 (3.9)
	Central and lateral	4 (3.9)

**Table 2:** Neck LN metastasis level.

Level	Prevalence
II	2/24 (8.3%)
III	4/24 (16.7%)
IV	8/24 (33.3%)
V	0/24 (0%)
VI	20/24 (83.3%)

**Table 3:** Patients with multifocal disease.

Patient number	Tumor foci diameter (mm)	Total size (mm)	Central LN	Lateral LN	Central and lateral LN	ETE	Suspicious foci seen at preop U/S
1	10, 2	12	+	−	−	−	1/2
2	9, 5	14	−	−	−	+	2/2
3	9, 4	13	−	+	−	+	2/2
4	9, 4	13	−	−	−	−	2/2
5	8, 5	13	−	−	−	−	2/2
6	8, 4	12	−	−	−	−	2/2
7	8, 2	10	+	−	−	+	2/2
8	6, 6, 5, 4	21	−	−	−	+	3/4
9	6, 5	11	−	−	−	−	2/2
10	6, 5	11	−	−	−	+	2/2
11	6, 5	11	−	−	−	−	2/2
12	6, 4, 3	13	−	−	+	−	3/3
13	6, 4	10	+	−	−	−	2/2
14	5, 4	9	+	−	−	+	2/2
15	5, 4	9	−	−	−	−	2/2
16	5, 2	7	−	−	−	−	2/2
17	4, 4	8	−	−	−	−	2/2
18	4, 4	8	+	−	−	+	2/2
19	4, 4	8	−	−	+	−	1/2
20	4, 2	6	−	−	−	+	1/2
21	4, 2.8	6.8	−	−	+	−	2/2
22	3, 1	4	−	−	−	−	1/2

+: present. −: absent.

**Table 4:** Association between different characteristics and lymph node metastasis.

Characteristics	Categories	Percentage of LN metastasis	*p* value^†^	Odds ratio (95% confidence interval)
Gender	Male	23.8	0.97	1.02 (0.33, 3.15)
	Female	23.5		Ref
Age	<55	24.1	0.82	1.14 (0.37, 3.48)
	≥55	21.7		Ref
Family history	Yes	20.0	0.73	0.79 (0.20, 3.05)
	No	24.1		Ref
Multifocality	Yes	40.9	**0.03**	3.00 (1.08, 8.30)
	No	18.8		Ref
Hashimoto's thyroiditis	Yes	12.5	0.08	0.36 (0.11, 1.15)
	No	28.6		Ref
Graves' disease	Yes	33.3	0.56	1.65 (0.14, 19.06)
	No	23.2		Ref
Tumor size (mm)			**0.05** ^**¶**^	**1.23 (1.00, 1.52)**
Tumor size	≤5 mm	17.0	0.15	Ref
	>5 mm	29.1		2.00 (0.77, 5.21)
Total tumor size (mm)			**0.03** ^**¶**^	**1.19 (1.02, 1.40)**
Total tumor size	≤5 mm	14.6	0.07	Ref
	>5 mm	30.0		2.50 (0.90, 6.98)
ETE	Yes	50.0	**0.00**	6.40 (2.36, 17.33)
	No	13.5		Ref

^†^ *p* value for the *χ*^2^ test or Fisher's exact test.^¶^*p* value of univariable logistic regression.

**Table 5:** Association between different characteristics and ETE.

Characteristics	Categories	Percentage of ETE	*p*value^†^	Odds ratio (95% confidence Interval)
Gender	Male	23.8	0.68	0.79 (0.26, 2.40)
	Female	28.4		Ref
Age	<55	27.8	0.87	1.09 (0.38, 3.14)
	≥55	26.1		Ref
Family history	Yes	33.3	0.58	1.39 (0.43, 4.50)
	No	26.4		Ref
Multifocality	Yes	36.4	0.29	1.71 (0.63, 4.69)
	No	20.0		Ref
Hashimoto's thyroiditis	Yes	25.0	0.71	0.83 (0.32, 2.16)
	No	28.6		Ref
Graves' disease	Yes	66.7	0.18	5.62 (0.49, 64.54)
	No	26.3		Ref
Tumor size (mm)			**0.01** ^**¶**^	**1.29 (1.05, 1.59)**
Tumor size	≤5 mm	19.1	0.08	Ref
	>5 mm	34.5		2.23 (0.89, 5.56)
Total tumor size (mm)			**0.04** ^**¶**^	**1.18 (1.01, 1.38)**
Total tumor size	≤5 mm	17.1	0.05	Ref
	>5 mm	34.4		2.55 (0.97, 6.73)
LN metastasis	Yes	58.3	**0.00**	
	No	17.9		

^†^ *p* value for the *χ*^2^ test or Fisher's exact test. ^¶^*p* value of univariable logistic regression.

**Table 6:** Prevalence and odds ratios (and 95% confidence interval) of LN metastasis and ETE by tumor size (≤5 mm, >5 mm), stratified by multifocality.

	Unifocal			Multifocal			
	≤5 mm	>5 mm		≤5 mm	>5 mm		
	Prevalence	Prevalence	Odds ratio (95% CI)	Prevalence	Prevalence	Odds ratio (95% CI)	*p* value^†^
LN metastasis	10.5	26.2	3.02 (0.87, 10.46)	44.4	38.5	0.78 (0.14, 4.39)	**0.03**
ETE	15.8	33.3	2.67 (0.90, 7.87)	33.3	38.5	1.25 (0.21, 7.41)	0.10

^†^ *p* value for interaction between tumor size and multifocality.

## Data Availability

The clinical study data used to support the findings of this study are included within the article.
